# Damp housing conditions as a determinant of psychological distress: a longitudinal analysis of the British Household Panel Survey

**DOI:** 10.1093/aje/kwaf263

**Published:** 2025-11-21

**Authors:** Maria Rosa Gatto, Ang Li, Erika Martino, Rebecca Bentley

**Affiliations:** NHMRC Centre of Research Excellence in Healthy Housing, Centre for Healthy Policy, Melbourne School of Population and Global Health, Faculty of Medicine, Dentistry and Health Sciences, The University of Melbourne, Parkville, VIC 3010, Australia; NHMRC Centre of Research Excellence in Healthy Housing, Centre for Healthy Policy, Melbourne School of Population and Global Health, Faculty of Medicine, Dentistry and Health Sciences, The University of Melbourne, Parkville, VIC 3010, Australia; NHMRC Centre of Research Excellence in Healthy Housing, Centre for Healthy Policy, Melbourne School of Population and Global Health, Faculty of Medicine, Dentistry and Health Sciences, The University of Melbourne, Parkville, VIC 3010, Australia; NHMRC Centre of Research Excellence in Healthy Housing, Centre for Healthy Policy, Melbourne School of Population and Global Health, Faculty of Medicine, Dentistry and Health Sciences, The University of Melbourne, Parkville, VIC 3010, Australia

**Keywords:** dampness, environmental exposures, mental health, housing, fixed effects, longitudinal study

## Abstract

Limited evidence exists regarding whether damp housing contributes to psychological distress. This study aimed to quantify the relationship between damp housing exposure and psychological distress. Data from the British Household Panel Survey (1996-2008) were used to assess the effect of damp housing on psychological distress in British households (*n* = 9189 at baseline). Indoor dampness exposure was measured using multiple indicators (condensation, leaky roof, rot, and damp walls/floors) and a measure of severity that quantified the number of exposures. Psychological distress was measured using a binary variable derived from the General Health Questionnaire. Multivariate fixed effects logistic regression models analyzed the hypothesized associations. Exposure to damp housing was associated with increased odds of psychological distress (OR, 1.09; 95% CI, 1.05-1.14; *P* < .01). Condensation was the strongest predictor (OR, 1.09; 95% CI, 1.03-1.13; *P* < .01). With each additional dampness indicator, odds of psychological distress increased by 4% (OR, 1.04; 95% CI, 1.02-1.07; *P* < .01). Among combinations of dampness indicators, the strongest association was for condensation and rot in windows/floors (OR, 1.25; 95% CI, 1.11-1.40; *P* < .01). These findings suggest damp housing exposure may increase the risk of psychological distress. Further research should investigate underlying mechanisms.

## Introduction

Physical housing conditions are recognized as an important and persistent contributor to psychological distress.^1^ Across high income nations, 10% to 50% of homes are estimated to be affected by dampness and mold.^2^ Damp housing and mold exposure is associated with adverse health outcomes, including asthma,^3^ respiratory infections,^4^ and rhinitis,^5^ especially in children.^6^ With the recent death of a child reported from exposure to mold in social housing,^7^ residential dampness and mold are gaining increasing attention as a preventable source of health harm in the United Kingdom and internationally.^8‑11^.

 While studies have identified associations between exposure to dampness and/or mold and depression, anxiety, stress, and reduced psychological wellbeing,^12‑16^ they are predominantly cross-sectional, generating associational evidence with limited control for confounding and reverse causation (the extent to which psychological distress leads to poorer living conditions).^17^ Longitudinal research is therefore required to strengthen the current body of evidence and accurately identify and quantify mental health effects of damp housing. Analysis of longitudinal data allows researchers to analyze the impact of becoming exposed over time, rather than analyzing the effect of contemporaneous exposure.^18^ Additionally, longitudinal analyses account for the issues inherent to cross-sectional studies by accounting for individual differences, time-invariant confounding, and observed time-varying confounding, therefore providing more reliable estimates of the true effect.^18^

Existing evidence suggests possible mechanisms through which indoor dampness may contribute to psychological distress. Given the impact of damp and mold-affected housing on respiratory health and allergies,^19^ and the mental health toll observed in people with those conditions,^20^^,^^21^ it is likely that knowing the potential effects of such exposure may induce stress and anxiety. Researchers have also hypothesized that fungal and mycotoxin exposure, sequelae of indoor dampness, may have neurotoxic or inflammatory effects,^12^^,^^22^ though this observation with regard to mold inhalation has only been observed in animal and cell models.^23^^,^^24^ Psychological distress in damp homes may also be a product of psychosocial stressors, including dissatisfaction with one's home, lack of control over housing conditions, social isolation due to reluctance to host people in the home, the need for essential housing maintenance, and financial concerns.^12^^,^^14^^,^^15^ However, these mechanisms remain speculative.

In this study, we used twelve waves (collected annually) of population-based, longitudinal data in the United Kingdom to analyze the effect of damp housing exposure on psychological distress. We used fixed effects models to assess how changes in a person's exposure over time affects their likelihood of reporting psychological distress, with several sensitivity analyses conducted to assess robustness. Specifically, our aim was to determine whether: (1) there is an effect of damp housing on psychological distress, (2) this effect varies depending on the type of exposure, and (3) if the effect is greater with greater severity of exposure.

## Methods

### Data source and study population

Data were drawn from the British Household Panel Survey (BHPS). The BHPS is a nationally representative household-based longitudinal study that followed more than 10 000 individuals 15 years or older from 5000 households between 1991 and 2009.^25^ An additional 1500 households from Scotland and Wales (in 1999) and 2000 households from Northern Ireland (in 2001) were added to increase the sample size.^25^ The present analysis was conducted on the data collected annually from waves 6 to 18 (1996 to 2008). Data were collected via interviews and self-completion questionnaires. The analytical sample consisted of individual respondents 15 years and older, who were followed for at least two consecutive waves. The main analytical sample contained 186 389 person-year observations, with the number of individuals per wave ranging between 9189 and 18 392.

### Psychological distress

Psychological distress was measured using the General Health Questionnaire (GHQ). The GHQ is a screening tool for general mental health, comprised of 12 items covering symptoms of depression and anxiety.^26^ For the purposes of this study, the bimodal form of the GHQ was used, wherein the responses to each of the twelve questions are recoded to 0 or 1 before summing them. Responses of “less than usual” or “no more than usual” were coded as 0, and responses of “rather more than usual” or “much more than usual” were coded as 1. This results in a scale ranging from 0 (no/little psychological distress) to 12 (high psychological distress).^26^ A cutoff score of 3 was used to create a binary indicator, with a score of 3 or more coded as symptomatic of psychological distress. A threshold of 2/3 points is commonly used for determining psychological distress.^27‑30^ However, determining GHQ cutpoints is complex, and Goldberg et al.^29^ recommend using the mean GHQ score as a rough guide to determining the threshold. In our sample, the mean GHQ score was 2.55, which translates to a cutpoint of 2/3 according to Goldberg et al.'s^29^ recommendations for non-clinical populations.

### Damp housing measures

Exposure to dampness was measured using four indicators of residential dampness: condensation; leaky roof; damp walls and/or floors; rot in windows and/or floors. These housing conditions were measured every wave from wave 6. We created a binary yes/no variable, defined as answering “yes” to any of the above. Additionally, a variable was created to describe severity of dampness, coded as the number (between 0 and 4) of dampness indicators reported. A third exposure variable captured all combinations of dampness indicators (eg, condensation only, condensation and leaky roof).

### Covariates

Demographic, health, and housing covariates were controlled for. Individual covariates included age (in years), sex (male/female), annual net household income (in GBP), country of residence (England/Wales/Scotland/Northern Ireland), highest qualification (degree/other higher degree/A-level or equivalent/GCSE or equivalent/other qualification/no qualification), employment status (paid employment/unemployed/self-employed/retired/student, apprentice, or trainee/maternity leave/family care or home/long-term sick or disabled/other), and long-term health conditions (no/yes), defined as reporting one of the following health conditions: problems or disability connected with arms, legs, hands, feet, back, or neck (including arthritis and rheumatism); difficulty in seeing, excluding, needing reading glasses; difficulty hearing; skin conditions and allergies; chest or breathing problems, including asthma and bronchitis; heart/high blood pressure or blood circulation problems; stomach/liver/kidney or digestive problems; diabetes; psychiatric conditions; alcohol or drug related problems; epilepsy; migraine or frequent headaches; cancer; stroke; other health problems. Household type (single non-elderly/single elderly/couple: no children/couple: dependent children/couple: non-dependent children/lone parent: dependent children/lone parent: non-dependent children/2+ unrelated adults/other households), residential tenure (owned outright/owned or being bought on mortgage/shared ownership (part owned, part rented)/rented/rent-free), and lack of adequate home heating (no/yes).

### Missing data

Data were missing for most variables (mostly less than 3.4%) (Table S1). The distribution of covariates was compared between participants with and without missing observations to determine whether missing data were associated with sociodemographic characteristics. Participants with missing data more frequently reported lower education level, lower rates of residency in England, and lower rates of paid employment (Table S2). Multiple imputation (MI) using chained equations with 50 imputations was performed to optimize the validity of the findings. The imputation models included all variables in the target analysis.

### Statistical analysis

Descriptive statistics were generated for each variable at baseline, with mean and SD for continuous variables and number and percentage for categorical variables reported. Transition probabilities between two consecutive waves for the exposure were also calculated to indicate how much change occurs in exposure over time.

**Figure 1:**
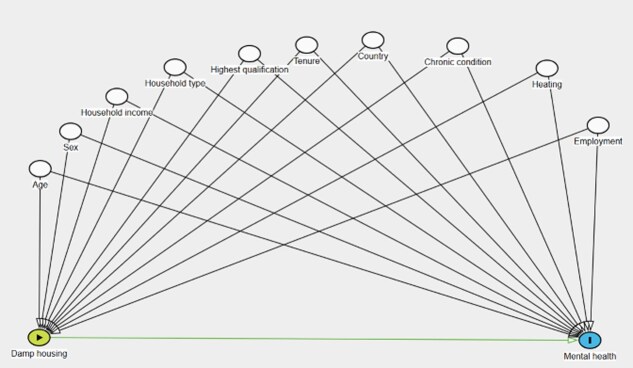
Directed acyclic graph of the association between damp housing conditions and psychological distress.

The theorized relationships between damp housing, psychological distress, and covariates are presented in the directed acyclic graph (Figure 1).

All analyses were conducted in Stata SE 18 (College Station, Texas). To remove time invariant confounding from characteristics, such as ethnicity, gender, and education (given the age of the sample), fixed effects logistic regression was used. Time-varying characteristics, such as income and adequacy of heating source, were adjusted as confounders. We then analyzed the effect of each dampness indicator available in the data individually and in combination (16 total combinations).

### Sensitivity analyses

A series of sensitivity analyses were completed to test the robustness of the analyses as follows:

1. A negative control outcome was selected that was not expected to be associated with exposure to damp housing—diabetes. Diabetes status was defined by how respondents answered the question: “Do you have any of the health problems or disabilities listed on this card?” Listed among the response options to this question was diabetes.
2. The exposure and outcome were swapped to see if there was a bidirectional association between psychological distress and damp housing and to gauge the size of this effect.
3. The sample was restricted to respondents who did not move home during the study period to reduce confounding from any unmeasured changes in home environments.
4. The effect was analyzed in a subsample of respondents who never reported a mental health condition prior to exposure.
5. The sensitivity of the multiple imputation model was tested by running the analysis on the non-imputed dataset
6. The association was analyzed in a more recent cohort, the UK Household Longitudinal Survey (UKHLS) available between 2012 and 2021. In this cohort, the question of residential dampness exposure was restricted to homes with no dependent children and at least one resident of pensionable age, giving a less representative population. Nonetheless, to see how the association varied between the older and newer cohorts, the overall fixed effects analysis, adjusted for the same covariates, was run for the UKHLS sample.

## Results

### Descriptive results

Table 1 shows the baseline (wave 6) descriptive statistics for our sample (*n* = 9189). At baseline, 29% of respondents reported at least one of the dampness indicators. 18% reported condensation, 4% a leaky roof, 9% damp walls and/or floors, and 12% rot in the windows and/or floor. Of those reporting exposure to dampness, most (71%) reported only one indicator of dampness, and less than 1% (*n* = 52) respondents reported all 4 indicators. The majority of respondents (72%) reported low/no psychological distress at baseline.

**Table 1:** Baseline (wave 6, 1996) characteristics of adults in the British Household Panel Survey aged 16+ years living in the United Kingdom.

**Variables**	***N* (9189)**	**%**
Mental health		
0-2 (Good)	6641	72.27
3+ (Poor)	2548	27.73
Overall dampness		
No	6519	70.94
Yes	2670	29.06
Condensation		
No	7500	81.62
Yes	1689	18.38
Leaky roof		
No	8820	95.98
Yes	369	4.02
Damp walls, floor		
No	8378	91.17
Yes	811	8.83
Rot in windows, floor		
No	8069	87.71
Yes	1120	12.19
Number of damp indicators		
0	6519	70.94
1	1710	18.61
2	653	7.11
3	255	2.78
4	52	0.57
Combination of damp indicators		
No damp indicators	6519	70.94
Condensation only	882	9.60
Rot only	492	5.35
Condensation and rot	258	2.81
Condensation and damp walls/floors	223	2.43
Damp walls/floors only	203	2.21
Condensation, damp walls/floors, and rot	179	1.95
Leaky roof only	133	1.45
Damp walls/floors and rot	64	0.70
Leaky roof and damp walls/floors	41	0.45
Condensation and leaky roof	38	0.41
Condensation, leaky roof and damp walls/floors	30	0.33
Leaky roof and rot	29	0.32
Condensation, leaky roof and rot	27	0.29
Leaky roof, damp walls/floors, and rot	19	0.21
All damp indicators	52	0.57
Age, mean, years	43.65 ± 18.46	
Household annual net income, mean (£)	19016.06 ± 11857.53	
Sex		
Male	4250	46.25
Female	4939	53.75
Household type		
Single non-elderly	594	6.46
Single elderly	684	7.44
Couple no children	2688	29.25
Couple: dep children	2840	30.91
Couple: non-dep children	1162	12.65
Lone par: dep children	398	4.33
Lone par: non-dep children	336	3.66
2+ Unrelated adults	326	3.55
Other households	161	1.75
Highest qualification		
Degree	949	10.33
Other higher degree	756	8.23
A-level or equivalent	1806	19.65
GCSE or equivalent	2337	25.43

**Table 1:** Continued

Variables	*N* (9189)	%
Other qualification	1014	11.03
No qualification	2327	25.32
Tenure		
Owned outright	6540	71.17
Owned/being bought on mortgage	42	0.46
Shared ownership (part owned/rented)	2451	26.67
Rented	142	1.55
Rent free	14	0.15
Country		
England	7915	86.14
Wales	474	5.16
Scotland	800	8.71
Northern Ireland	0	0
Long-term illness/condition		
No	3870	42.12
Yes	5319	57.88
Lack of adequate heating		
No	8544	92.98
Yes	645	7.02
Employment status		
Paid employment	4642	50.52
Unemployed	379	4.12
Self-employed	677	7.37
Retired	1633	17.77
Student/apprentice/trainee	690	7.51
Maternity leave	34	0.37
Family care/home	809	8.80
LT sick or disabled	317	3.45
Other	8	0.09
Diabetes		
No	8992	97.86
Yes	197	2.14
Mental health condition		
No	8576	93.33
Yes	613	6.67
Moved across waves 6-18		
No	4947	53.84
Yes	4242	46.16

Descriptive statistics are based on multiply imputed data—the estimates were derived from a randomly selected imputation (number 23).

Table 2 shows the transition probabilities for damp exposure. Unexposed respondents had a 10% likelihood of becoming exposed between waves, while exposed respondents had a 54% chance of remaining exposed.

**Table 2:** Transition probabilities for cross-wave dampness exposure (reporting any of condensation, leaky roof, damp walls/floors, and/or rot in windows/floors) between consecutive waves of the British Household Panel Survey, waves 6-18.

	Damp	
Damp	No	Yes
No	90.01	9.99
Yes	46.32	53.68

Table 3 shows the transition probabilities for psychological distress. People reporting low/no psychological distress had a 17% chance of reporting psychological distress in the following wave, while people reporting psychological distress had a 48% chance of reporting low/no psychological distress in the following wave.

**Table 3:** Transition probabilities for cross-wave changes in GHQ score between consecutive waves of the British Household Panel Survey, waves 6-18.

	GHQ score	
GHQ score	Below 3	3 or more
Below 3	82.87	17.13
3 or more	47.74	52.26

### Analytical results: effect of damp housing on psychological distress

The univariate fixed effects logistic regression analysis showed 10% increased odds (OR, 1.10; 95% CI, 1.06-1.15; *P* < .01) of psychological distress for damp housing-exposed individuals compared with their counterparts (Table 4). In the multivariate model, there was a slight attenuation of this estimate to 9% (Table 4).

**Table 4:** Unadjusted and adjusted fixed effects logistic regression estimates of the effect of damp housing exposure on the odds of psychological distress in the British Household Panel Survey, waves 6-18.

Damp	OR	95% CI	*P*
Unadjusted	1.10	1.06-1.15	<.01
Adjusted	1.09	1.05-1.14	<.01

Adjusted model includes adjustment for age, sex, household type, highest qualification, residential tenure, household annual net income, country of residence, chronic health conditions, lack of adequate heating, and employment status.

Comparison of dampness indicators (Table 5) showed that the strongest association was observed for condensation (OR, 1.09; 95% CI, 1.03-1.13; *P* = .001). There was no evidence of an effect on psychological distress of reporting a leaky roof (OR, 1.00; 95% CI, 0.92-1.09; *P* = .96).

**Table 5:** Comparison of odds ratios for the association between damp housing exposure and psychological distress by dampness indicator in the British Household Panel Survey, waves 6-18.

Dampness indicator	OR	95% CI	*P*
Condensation	1.09	1.03-1.13	<.01
Leaky roof	1.00	0.92-1.09	.96
Damp walls, floor, etc.	1.07	1.01-1.14	.02
Rot in windows, floors, etc.	1.06	1.00-1.13	.07

Models include adjustment for age, sex, household type, highest qualification, residential tenure, household annual net income, country of residence, chronic health conditions, lack of adequate heating, and employment status.

The odds of psychological distress increased by 4% with each additional dampness indicator (OR, 1.04; 95% CI, 1.02-1.07; *P* = .001) (Table 6).

**Table 6:** OR for the effect of an increase of 1 in the number dampness indicators reported on psychological distress in the British Household Panel Survey, waves 6–18.

	OR	95% CI	*P*
Number of dampness indicators	1.04	1.02-1.07	<.01

Models include adjustment for age, sex, household type, highest qualification, residential tenure, household annual net income, country of residence, chronic health conditions, lack of adequate heating, and employment status.

When comparing different combinations of dampness indicators (Table 7), the strongest associations were observed for: damp walls and/or floors (OR, 1.12; 95% CI, 1.02-1.23; *P* = .02), condensation (OR, 1.08; 95% CI, 1.02-1.16; *P* = .02), condensation and rot (OR, 1.25; 95% CI, 1.11-1.40; *P* < .01), and condensation and damp walls and/or floors (OR, 1.16; 95% CI, 1.05-1.29; *P* = .004).

**Table 7:** Odds ratios for the effect of different combinations of dampness indicators on psychological distress in the British household panel survey, waves 6-18.

Dampness indicator/s	OR	95% CI	*P*
Rot in windows/floors only	1.04	0.95-1.15	.36
Damp walls, floors only	1.12	1.02-1.23	.02
Leaky roof only	1.11	0.99-1.25	.08
Condensation only	1.08	1.02-1.16	.02
Damp walls, floors and rot in windows/floors	1.11	0.91-1.36	.29
Leaky roof and rot in windows/floors	0.96	0.71-1.29	.77
Leaky roof and damp walls/floors	1.02	0.83-1.26	.85
Condensation and rot	1.25	1.11-1.40	<.01
Condensation and damp walls/floors	1.16	1.05-1.29	.004
Condensation and leaky roof	0.80	0.62-1.04	.10
Condensation, damp, and rot	1.05	0.91-1.22	.49
Leaky roof, damp, and rot	0.98	0.70-1.35	.89
Condensation, leaky roof, and rot	1.40	0.95-2.09	.09
Condensation, leaky roof, and damp	1.03	0.92-1.28	.09
All damp indicators	0.99	0.81-1.21	.93

Models include adjustment for age, sex, household type, highest qualification, residential tenure, household annual net income, country of residence, chronic health conditions, lack of adequate heating, and employment status.

### Sensitivity analysis

When testing the robustness of our findings by estimating the effect of damp housing exposure on the negative control outcome of diabetes, the negative outcome control was not found to be associated with exposure to damp (OR, 1.02; 95% CI, 0.81-1.29; *P* = .87), supporting that the observed relationships in these models are not severely biased (Table S3).

When the exposure and outcome were swapped, that is, estimating the effect of psychological distress on damp housing (Table S4), a bidirectional association was observed (OR, 1.09; 95% CI, 1.05-1.14; *P* < .01), reinforcing the benefit of using longitudinal data to model changes in exposure and thereby account for reverse causation.

When restricting the analysis to people who remained in the same dwelling within the period under study, no significant difference in the effect estimate was observed compared with the overall sample (OR, 1.09; 95% CI, 1.02-1.17; *P* = .01) (Table S5).

When restricting the analyses to those who had not reported a mental health condition prior to exposure, the effect attenuated minimally however remained significant (OR, 1.07; 95% CI, 1.01-1.13; *P* = .01) (Table S6).

To test the sensitivity of the multiply imputed dataset, we ran the overall logistic regression analysis on the non-imputed dataset. The imputed dataset produced a slightly greater odds ratio (OR, 1.07; 95% CI, 1.02-1.11; *P* = .002), though the difference in effect estimates was minimal, suggesting that the imputed data had accounted for bias from missing variables (Table S7).

As the final sensitivity analysis, the association was examined in a newer cohort study (UKHLS) restricted to homes with no dependent children and at least one adult of pensionable age. In this cohort (*n* = 8414, Table S8), 11% increased odds of psychological distress were reported with exposure to damp housing after adjustment covariates (95% CI, 0.98-1.27; *P* = .11), which was consistent with the primary analysis in our broader cohort. While the point estimate remained similar, the confidence interval crossed the null, likely due to reduced statistical power from the restricted sample. Overall, the results support the robustness of the observed association across different study populations.

## Discussion

Our findings provide evidence for an association between exposure to damp housing and increased odds on psychological distress. This finding was observed in both the main analysis and in extensive sensitivity analyses. Visible condensation appears to be a significant driver of this relationship, with the strongest associations found for condensation overall, and for a combination of condensation and observed rot in windows and/or floors.

These findings align with the findings of cross-sectional studies of adult populations that identify dampness and mold as housing-related risk factors for psychological distress, reporting associations with depression,^12^ anxiety,^31^ stress,^32^ psychological distress,^15^ and subjective mental health.^13^^,^^16^^,^^33^ Importantly, our current study offers more causally robust estimates based on longitudinal data that better address the effects of confounding.

The effects on psychological distress observed in our study may be explained by several factors. First, exposure to dampness is associated with a range of physical health effects, including asthma and allergic rhinitis.^2^ Both asthma and allergic rhinitis are associated with depression and anxiety, likely due to the stress of the symptoms themselves, as well as the mental burden of managing the symptoms and triggers.^20^^,^^21^ Similarly, there could be an added physical, psychological and financial burden of managing the dampness (as the asthma trigger) to reduce asthma exacerbations that may explain the observed association.^34^ In addition, other psychosocial mechanisms may be at play. Whether or not a home has signs of dampness is associated with decreased housing satisfaction,^35^ which in itself may be a predictor of mental health.^36^ Financial concerns may be an additional explanatory factor: households reporting dampness may report more problems affording necessary utilities and maintenance costs such as heating the home and rectifying causes of dampness, which in turn may affect mental health due to the stress of financial strain.^14^^,^^37^^,^^38^ The potential causal mechanisms remain theoretical, with more research needed to identify and investigate these pathways.

This study has several key strengths. First, large, nationally representative longitudinal data was used, where each variable was measured at every wave. The use of fixed effects models in the analysis allowed us to account for unmeasured individual-level time-invariant confounding, thereby reducing bias and enabling a more accurate effect estimate.^18^ Additionally, these models allowed us to analyze the effects of damp housing exposure on psychological distress within people, showing the effect of transitioning to an exposed state. The use of multiple imputation to account for missing data reduced this potential bias. This study also contributes to the empirical evidence on the mental health effects of damp housing by identifying the primary type of exposure driving these effects and examining the role of exposure severity.

However, our study also has several limitations. First, the data used concluded in 2008, which may limit generalizability to the current population, given transitions in some sociodemographic characteristics. For example, residential tenure, and shared ownership is less common today, and rates of mortgage-holding and renting have increased—therefore, residential tenure may have a different confounding effect on the association. However, our sensitivity analysis of more contemporaneous data suggests this may not be a significant issue. Second, all measures are self-reported, which may introduce reporting bias. For example, people with poorer mental health may also have reported damp housing at higher rates than people with good mental health, which was shown in the second sensitivity analysis. There are also some limitations associated with the use of fixed effects model. The effect of persistent damp housing on psychological distress is an estimation, since only observations where the exposure varied contributed to the fixed effects estimates.^18^ Additionally, despite our robust modeling approach, several sources of bias remain including the effect of unmeasured time-varying confounders and differential attrition from the longitudinal study of people based on their exposure and/or outcomes status. Finally, the survey did not include information about the environmental and climatic conditions at the time of the survey (a key predictor of a home becoming damp). We therefore could not account for these as a source of confounding in the model.

The findings of this study highlight the need for more research into the mental health impacts of damp- and mold-affected housing, as well as physical housing conditions. In particular, the finding of an association between housing dampness and psychological distress warrants investigation of causal pathways and vulnerable subpopulations, such as those with respiratory conditions. Identification and analysis of causal pathways and vulnerable populations would enable a deeper understanding of why such associations exist and help to justify and target interventions aimed at improving health by remediating housing. Future studies would also benefit from data on environmental conditions, both in terms of climate and physical housing conditions, at the time of the survey to reduce residual confounding by these factors.

The results of this study have important clinical and policy implications. While more research is needed to further elucidate the association and identify its primary drivers, the existence of such an association emphasizes the need to include questions on housing conditions such as dampness and mold when evaluating patients with mental health concerns. Given that the causes of mental health conditions, such as depression and anxiety, are multifactorial, it is vital to address environmental determinants of mental health in treatment plans, in addition to medication and psychotherapy. Evidence from housing condition intervention studies suggest that improvements to housing conditions, including insulation, ventilation, mold removal, and heating have positive effects on mental health.^39‑42^ In cases where dampness is identified as a stressor, clinicians should consider acting as advocates for their patients, particularly where a patient is in rented or social housing and has limited control over their housing conditions. The present study also adds to increasing evidence supporting calls for better standards, regulations, and policies to address housing conditions. Investing in housing remediation to address dampness and mold may have effects beyond housing value and physical health—the results of this study suggest that remediating dampness can alleviate or ameliorate psychological distress by removing a source of stress and anxiety.

## Supplementary Material

Web_Material_kwaf263

## Data Availability

Original data from the British Household Survey and the UK Household Longitudinal Study were collected by the University of Essex, Institute for Social and Economic Research. Data is available for download from the UK Data Service.
