# Supplementary material for: Damp housing conditions as a determinant of psychological distress: a longitudinal analysis of the British Household Panel Survey
Source: Am J Epidemiol. 2025 Nov 21;195(5):1284–91. doi: 10.1093/aje/kwaf263 (PMC13149023; doi:10.1093/aje/kwaf263)
Supplement: Web_Material_kwaf263 [file web_material_kwaf263.zip › AJE_SuppMaterial.docx]

**Supplementary Material**

**Damp housing conditions as a determinant of psychological distress: a longitudinal analysis of the British Household Panel Survey**

Maria Rosa Gatto, Ang Li, Erika Martino, and Rebecca Bentley

**Table S1.** Missing data summary – Page 2

**Table S2.** Comparison of covariates between individuals with/without missing data **–** Page 2

**Table S3.** Results of sensitivity analysis using negative control outcome (diabetes), adjusted for all covariates – Page 4

**Table S4.** Results of fixed effects logistic regression analysis for the association between mental health and damp housing – Page 4

**Table S5.** Fixed effects logistic regression analysis of the association between damp housing exposure and mental health in a subsample of participants who remained in the same home across all waves – Page 4

**Table S6.** Fixed effects logistic regression analysis of the association between damp housing exposure and mental health in a subsample of participants who did not report a mental health condition in any wave – Page 4

**Table S7.** Logistic regression analysis of the association between damp housing exposure and mental health using the non-imputed dataset – Page 5

**Table S8.** Unadjusted and adjusted fixed effects logistic regression analyses of the association between damp housing exposure and mental health using data from the UK Household Longitudinal Survey (UKHLS) – Page 5

**Supplementary Material**

**Table S1: Missing data summary (total person-years = 186,389)**

| **Variable** | **Person-years missing** | **% Missing** |
| --- | --- | --- |
| Highest qualification | 3,551 | 1.91 |
| Resident at present address last year | 2,570 | 1.39 |
| Sex | 81 | 0.04 |
| Health problems | 2,810 | 1.51 |
| Diabetes | 2,810 | 1.51 |
| Mental health condition | 2,810 | 1.51 |
| Age | 6 | <0.01 |
| Household type | 0 | 0 |
| GHQ score | 11,897 | 6.38 |
| Tenure | 3,654 | 1.96 |
| Lack of adequate heating | 3,654 | 3.36 |
| Condensation | 6,280 | 3.37 |
| Leaky roof | 6,386 | 3.43 |
| Damp walls, floors, etc. | 6,284 | 3.37 |
| Rot in windows, floors | 6,345 | 3.40 |
| Household annual net income | 5,225 | 2.80 |
| Country | 1,363 | 0.73 |
| Damp | 6,215 | 3.33 |

**Table S2: Comparison of covariates between individuals with/without missing data (person-years = 186,389)**

|  | **Complete observations (n = 150,138)** | **Observations with missing data (n =** |
| --- | --- | --- |
| **Covariates (continuous)** | **Mean (SD)** | **Mean (SD)** |
| Age (years) | 45.44 (18.39) | 46.29 |
| Household annual net income (£) | 23,804.71 (16,492.43) | 21,570.21 (14,715.66) |
| **Covariates (categorical)** | **Person-years (%)** | **Person-years (%)** |
| Sex |  |  |
| Male | 68,204 (45.43) | 16,276 (45) |
| Female | 81,934 (54.57) | 19,894 (55) |
| Household type |  |  |
| Single non-elderly | 10,087 (6.72) | 3,006 (8.29) |
| Single elderly | 11,331 (7.55) | 3,451 (9.52) |
| Couple no children | 45,010 (29.98) | 9,916 (27.35) |
| Couple: dep children | 45,688 (30.43) | 10,290 (28.39) |
| Couple: non-dep children | 19,034 (12.68) | 4,604 (12.70) |
| Lone par: dep children | 7,523 (5.01) | 1,848 (5.10) |
| Lone par: non-dep children | 5,771 (3.84) | 1,618 (4.46) |
| 2+ unrelated adults | 3,100 (2.06) | 769 (2.12) |
| Other households | 2,594 (1.73) | 749 (2.07) |
| Highest qualification |  |  |
| Degree | 19,381 (12.91) | 3,699 (11.31) |
| Other higher degree | 13,069 (8.70) | 2,616 (8.00) |
| A-level or equivalent | 32,457 (21.62) | 6,238 (19.08) |
| GCSE or equivalent | 36,983 (24.63) | 7,575 (23.17) |
| Other qualification | 15,479 (10.31) | 3,313 (10.13) |
| No qualification | 32,769 (21.83) | 9,259 (28.31) |
| Tenure |  |  |
| Owned outright | 110,309 (73.47) | 23,031 (70.65) |
| Owned/being bought on mortgage | 605 (0.40) | 105 (0.32) |
| Shared ownership (part owned/rented) | 36,812 (24.52) | 8,835 (27.10) |
| Rented | 2,036 (1.36) | 533 (1.64) |
| Rent free | 376 (0.25) | 93 (0.29) |
| Country |  |  |
| England | 86,840 (57.84) | 16,345 (46.85) |
| Wales | 23,065 (15.36) | 5,982 (17.15) |
| Scotland | 25,791 (17.18) | 6,238 (17.88) |
| Northern Ireland | 14,442 (9.62) | 6,323 (18.12) |
| Long-term illness/condition |  |  |
| No | 60,195 (40.09) | 13,387 (40.03) |
| Yes | 89,943 (59.91) | 20,054 (59.97) |
| Lack of adequate heating |  |  |
| No | 143,616 (95.66) | 28,513 (95.10) |
| Yes | 6,522 (4.34) | 1,470 (4.90) |
| Employment status |  |  |
| Paid employment | 76,766 (51.13) | 16,930 (46.75) |
| Unemployed | 4,968 (3.31) | 1,403 (3.87) |
| Self-employed | 10,133 (6.75) | 2,359 (6.51) |
| Retired | 30,699 (20.45) | 8,286 (22.88) |
| Student/apprentice/trainee | 9,212 (6.14) | 2,406 (6.64) |
| Maternity leave | 675 (0.45) | 132 (0.36) |
| Family care/home | 10,599 (7.06) | 2,608 (7.20) |
| LT sick or disabled | 6,406 (4.27) | 1,896 (5.24) |
| Other | 680 (0.45) | 191 (0.53) |

**Table S3: Results of sensitivity analysis using negative control outcome (diabetes), adjusted for all covariates**

|  | **Odds Ratio** | **95% Confidence Interval** | ***P*-value** |
| --- | --- | --- | --- |
| Damp |  |  |  |
| No | Ref |  |  |
| Yes | 1.02 | 0.81, 1.29 | 0.87 |

Note: Models include adjustment for age, sex, household type, highest qualification, residential tenure, household annual net income, country of residence, chronic health conditions, lack of adequate heating, and employment status.

**Table S4: Results of fixed effects logistic regression analysis for the association between mental health and damp housing**

|  | **Odds Ratio** | **95% Confidence Interval** | ***P*-value** |
| --- | --- | --- | --- |
| Mental health |  |  |  |
| Good | Ref |  |  |
| Poor | 1.09 | 1.05, 1.14 | <0.01 |

Note: Models include adjustment for age, sex, household type, highest qualification, residential tenure, household annual net income, country of residence, chronic health conditions, lack of adequate heating, and employment status.

**Table S5:** **Fixed effects logistic regression analysis of the association between damp housing exposure and mental health in a subsample of participants who remained in the same home across all waves**

|  | **Odds Ratio** | **95% Confidence Interval** | ***P*-value** |
| --- | --- | --- | --- |
| Damp |  |  |  |
| No | Ref |  |  |
| Yes | 1.09 | 1.02, 1.17 | 0.01 |

Note: Models include adjustment for age, sex, household type, highest qualification, residential tenure, household annual net income, country of residence, chronic health conditions, lack of adequate heating, and employment status.

**Table S6: Fixed effects logistic regression analysis of the association between damp housing exposure and mental health in a subsample of participants who did not report a mental health condition in any wave**

|  | **Odds Ratio** | **95% Confidence Interval** | ***P*-value** |
| --- | --- | --- | --- |
| Damp |  |  |  |
| No | Ref |  |  |
| Yes | 1.07 | 1.01, 1.13 | 0.01 |

Note: Presence of a mental health condition was measured every wave by a question asking participants to report whether they had anxiety, depression, or a psychiatric condition. Models include adjustment for age, sex, household type, highest qualification, residential tenure, household annual net income, country of residence, chronic health conditions, lack of adequate heating, and employment status.

**Table S7: Logistic regression analysis of the association between damp housing exposure and mental health using the non-imputed dataset**

|  | **Odds Ratio** | **95% Confidence Interval** | ***P*-value** |
| --- | --- | --- | --- |
| Damp |  |  |  |
| No | Ref |  |  |
| Yes | 1.07 | 1.02, 1.11 | 0.002 |

Note: models include adjustment for age, sex, household type, highest qualification, residential tenure, household annual net income, country of residence, chronic health conditions, lack of adequate heating, and employment status.

**Table S8: Unadjusted and adjusted fixed effects logistic regression analyses of the association between damp housing exposure and mental health using data from the UK Household Longitudinal Survey (UKHLS)**

|  | **Odds Ratio** | **95% Confidence Interval** | ***P*-value** |
| --- | --- | --- | --- |
| Unadjusted | 1.17 | 1.03, 1.33 | 0.02 |
| Adjusted | 1.11 | 0.98, 1.27 | 0.11 |

Note: models include adjustment for age, sex, household type, highest qualification, residential tenure, household annual net income, country of residence, chronic health conditions, lack of adequate heating, and employment status.
